# Large proportion of genes in one cryptic WO prophage genome are actively and sex-specifically transcribed in a fig wasp species

**DOI:** 10.1186/1471-2164-15-893

**Published:** 2014-10-13

**Authors:** Guan-Hong Wang, Li-Ming Niu, Guang-Chang Ma, Jin-Hua Xiao, Da-Wei Huang

**Affiliations:** Key Laboratory of Zoological Systematics and Evolution, Institute of Zoology, Chinese Academy of Sciences, Beijing, 100101 China; University of Chinese Academy of Sciences, Beijing, 100039 China; Environment and Plant Protection Institute, Chinese Academy of Tropical Agricultural Sciences, Danzhou, Hainan China; Plant Protection College, Shandong Agricultural University, Tai’an, 271018 China

**Keywords:** Defective prophage, Bacteriophage WO, Reverse-transcription PCR, Real-time quantitative PCR, Ankyrin-domain-containing protein

## Abstract

**Background:**

Cryptic prophages are genetically defective in their induction and propagation, and are simply regarded as genetic remnants. There are several putative cryptic WO prophages in the sequenced *Wolbachia* genomes. Whether they are lytic is unclear and their functions are poorly understood. Only three open reading frames (ORFs) in cryptic WO prophages have been reported to be actively transcribed.

**Results:**

In this study, we comprehensively examined the transcription of the only cryptic WO prophage (WOSol) in a *Wolbachia* strain that infects a fig wasp, *Ceratosolen solmsi* (Agaonidae, Chalcidoidea). By analyzing the transcriptions of all the ORFs of WOSol in both sexes of *C. solmsi*, using qualitative and quantitative methods, we demonstrated that i) a high percentage of ORFs are actively transcribed (59%, 17/29); ii) the expression of these ORFs is highly sex-specific, with a strong male bias (three in females and 15 in males); iii) an *ank* (ankyrin-domain-containing) gene actively transcribed in both wasp sexes is more highly expressed in males.

**Conclusions:**

A large proportion of the genes in the cryptic WO prophage WOSol are expressed, which overturns the concept that cryptic prophages are simply genetically defective. The highly sex-specific expression patterns of these genes in the host suggest that they play important roles in *Wolbachia* biology and its reproductive manipulation of its insect host, particularly through the males.

**Electronic supplementary material:**

The online version of this article (doi:10.1186/1471-2164-15-893) contains supplementary material, which is available to authorized users.

## Background

Bacteriophages, or phages, are viruses that infect bacteria, and can be either lytic or temperate. Lytic phages are strict pathogens of their bacterial hosts, and their infections culminate in the production of large numbers of new viral particles and lysis of the host cells. Temperate phages, such as the WO phages in *Wolbachia*, have two different forms. They can be lysogenic, with the viral DNA integrating into the host DNA and replicating as part of the host chromosome, which is referred to as the “prophage” form [1]. However, upon some signals, they can also be induced to produce a lytic form, which generates virions and causes bacterial lysis [2, 3]. For example, some WO prophages have been reported to form virions, including WOCauB2 and WOCauB3 [4], WOVitA1 [5, 6], WOCauB1 [7], and at least one haplotype located in the *Wolbachia* infecting *Drosophila melanogaster* [8].

*Wolbachia*, a cytoplasmically inherited Rickettsiales, causes a number of reproductive anomalies in its arthropod hosts, including cytoplasmic incompatibility (CI) [9], parthenogenesis [10], feminization of genetic males [11], and male killing [12]. These reproductive phenotypes impart a selective advantage on *Wolbachia* [13, 14], facilitating the spread of *Wolbachia* infections in the host population. More than 80% of *Wolbachia* strains contain bacteriophage-WO-related gene fragments [15], so whether the mobile genetic elements of the WO phages contribute to *Wolbachia*’s reproductive manipulation of their hosts is a hot topic. Based on evidence from G + C content and codon usage analyses of *Wolbachia* and WO, some scholars indicate that *Wolbachia* and WO have had a very long evolutionary association and that WO must confer some benefit on *Wolbachia* [16]. However, some *Wolbachia* strains without WO can still manipulate the reproduction of their hosts, indicating the dispensability of WO in the function of *Wolbachia* [15, 17].

Selective pressure can cause the degradation of prophages to genetically defective forms [18]. Prophages may become trapped in the chromosome of the host through recombination and/or deletion, and gradually decay [19], becoming inactive in terms of cell lysis, phage particle production, and plaque formation. These prophage fragments are referred to as cryptic or defective prophages [20]. To date, several putative cryptic WO prophages have been found in the sequenced *Wolbachia* strains [21, 22]. However, all of these putative cryptic WO prophages occur with at least one other complete WO prophage, carrying the complete head, baseplate, and tail gene modules that are essential for proper phage function [22, 23]. For example, prophages WORiA and WORiB are regarded as cryptic prophages in *Wolbachia w*Ri, which infects *D. simulans*, but occur with at least one active phage, WORiC [23].

Bacteriophages play many roles in the ecology and genomic evolution of bacteria. For example, they can mediate lateral gene transfer [24], and in some cases provide their hosts with beneficial genes [25, 26]. Bacteriophages can also regulate the numbers of their host bacteria by inhibiting their replication or inducing cell lysis [5]. Furthermore, as mentioned above, some WO phages may contribute to *Wolbachia*’s reproductive manipulation of their hosts. Cryptic prophages can also benefit their hosts, because they can be involved in the host physiology and biofilm formation [27], and can increase the host’s resistance to general environmental stresses and to antibiotics [28]. Although cryptic prophages may have functions in the host, we still know very little about the mechanisms of these functional processes. The introduction of novel genes by these phages may confer beneficial phenotypes on their hosts [28] and prophage–prophage interactions could also be important pathways through which the potential activities of defective prophages are induced [29].

However, the expression and functions of cryptic WO prophage in *Wolbachia* are still poorly known. Until now, only two *ank* genes [30] and a putative DNA adenine methyltransferase gene (*met2*) [23] located within the cryptic WO prophage WORiB have been reported to be actively transcribed, and may play active roles in *Wolbachia* biology [20]. This suggests that there is an extreme paucity of data on the active transcription of the genes of cryptic WO prophages. In this study, we confirmed a cryptic WO prophage, WOSol, in *Wolbachia* strain *w*Sol, which infects the fig wasp *Ceratosolen solmsi*. This is the only prophage detected in *w*Sol. WOSol is highly degenerate and may lack a tail module. We demonstrated a comprehensive analysis of the transcription of this putative cryptic phage WO. Surprisingly, we found that a high percentage of the genes of this cryptic prophage are actively transcribed and display significantly different expression patterns in female and male fig wasps.

## Results

### Only one cryptic prophage occurred in *C. solmsi*

In our previous study [31], we have demonstrated that the fig wasp species *C. solmsi* is infected by a single *Wolbachia* strain that contains only one defective prophage WOSol, which lacks a tail module. Here, using real-time quantitative PCR (real-time qPCR), we counted and compared the densities of the *Wolbachia* genomes (represented by the single-copy *groEL* gene), and the phage WOSol genomes (represented by the single-copy *orf7* gene) to determine whether WOSol was replicated extrachromosomally (the primers are listed in Additional file 1). With a single lysogenic copy of WOSol, the WOSol density should always equal (no lytic activity) or exceed (with lytic activity producing multiple phage virions) the *w*Sol copy number [32]. The correlation between the copy counts of *groEL* and *orf7* can thus reflect the total phage abundance in the female and male individuals of *C. solmsi*. We calculated the relative copy numbers (*orf7*:*groEL*) in 31 female and 35 male wasp individuals. The mean relative densities were consistent with the prediction of a single integrated copy in the *Wolbachia* genome and indicated no extrachromosomal WOSol (0.88 ± 0.05 in females and 1.15 ± 0.06 in males; all p values >0.05; two-tailed *t* test; Additional file 2).

**Figure 1:**
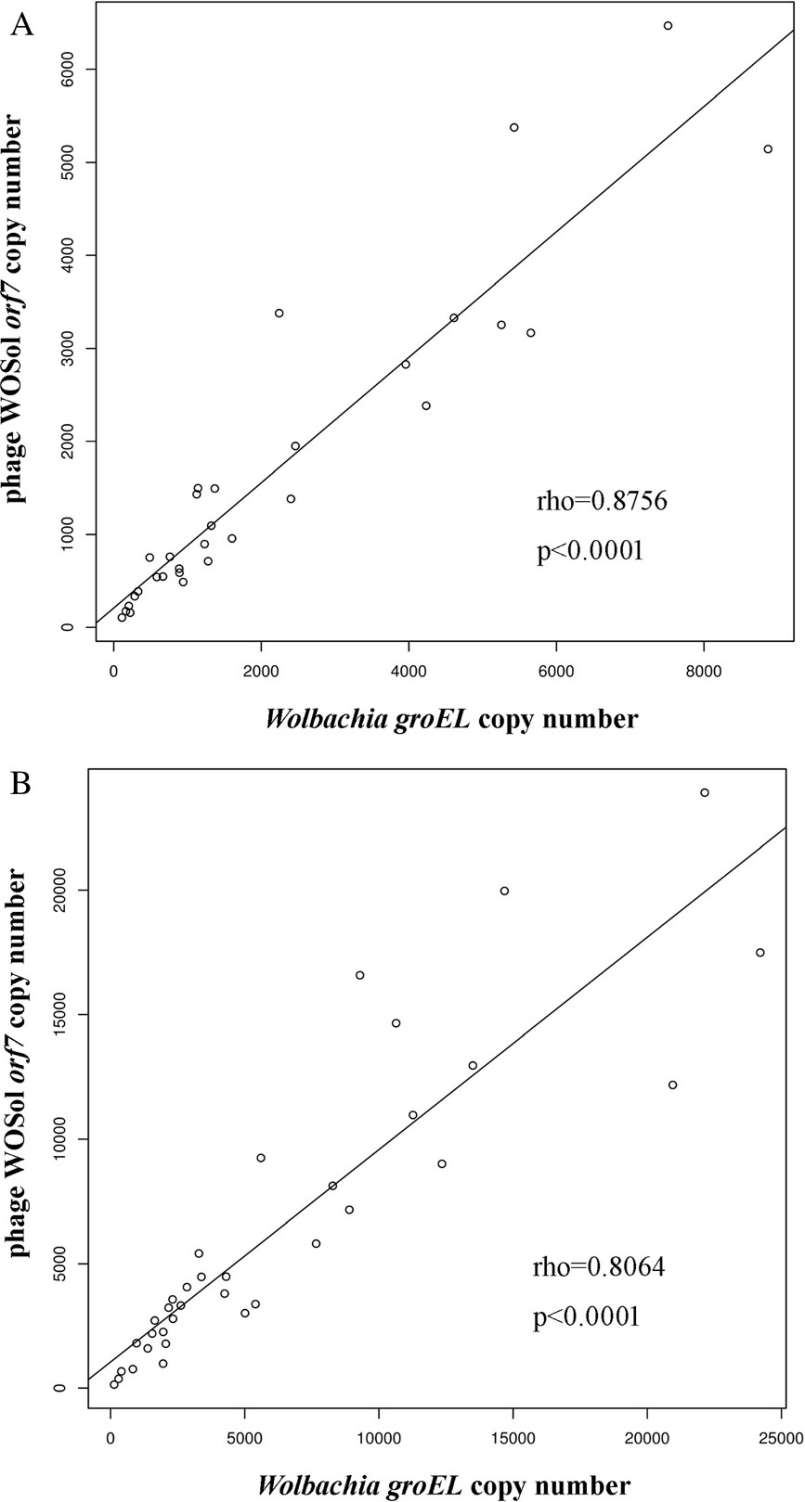
**The correlation between the abundance of prophage WOSol and that of** ***Wolbachia w*** **Sol in females (A) and males (B) of** ***C. solmsi*** **.** Each circle on the charts denotes the absolute copy number of a single-copy gene (*orf7*) of prophage WOSol (according to the vertical axis) and a single-copy gene (*groEL*) of *Wolbachia w*Sol (according to the horizontal axis) in an adult female or male *C. solmsi* individual infected with *Wolbachia*. Altogether, 31 female and 35 male wasps were investigated. Correlation coefficients (rho values) and significances (P values) were calculated according to the nonparametric method of Spearman’s rho.

The total bacteriophage WOSol abundance correlated strongly with the total bacterial abundance in both females (rho = 0.8756, P <0.0001; Figure 1A) and males (rho = 0.8064, P <0.0001; Figure 1B), as expected for a cryptic prophage with which a lysogenic phage is co-transmitted in the bacterial host.

We also designed degenerate PCR primers based on the sequenced phage WO genomes (phage tail tape measure protein, GenBank accession number: CP001391.1|:758319-759499, AB478515.1|:52906-54086, AB478516.1|:48642-49819, CP003883.1|:1115914-1117093, AE017196.1|:553968-555151, CP003884.1|:442584-443767, AM999887.1|:1409653-1410819; phage late control gene protein GpD, GenBank accession number: AB478515.1|:55418-56398, CP001391.1|:755998-756978, AB478516.1|:51163-52143, CP003883.1|:1118431-1119411, HQ906662.1|:38950-39741, AM999887.1|:482503-483235, AM999887.1|:1412374-1413106) to amplify the bacteriophage WO tail genes, and obtained no successful amplification with normal PCR (data not shown). This further suggested that WOSol had no tail module. Moreover, we amplified no WOSol genes from *Wolbachia*-uninfected fig wasp individuals with normal PCR, by which we could exclude the possibility that prophage WOSol was present in the genome of the fig wasp.

### High percentage of genes in the cryptic prophage WOSol genome were actively transcribed

Using reverse transcription PCR (RT–PCR) and nested RT–PCR, which have been commonly used in previous WO phage studies (details in the Methods section), we examined the mRNA expression of all 29 genes of the cryptic prophage WOSol in both female and male fig wasps (the primers are listed in Additional file 3). In females, only three ORFs were actively transcribed, whereas 15 ORFs were actively transcribed in males (Table 1 and Additional file 4).

**Figure 2:**
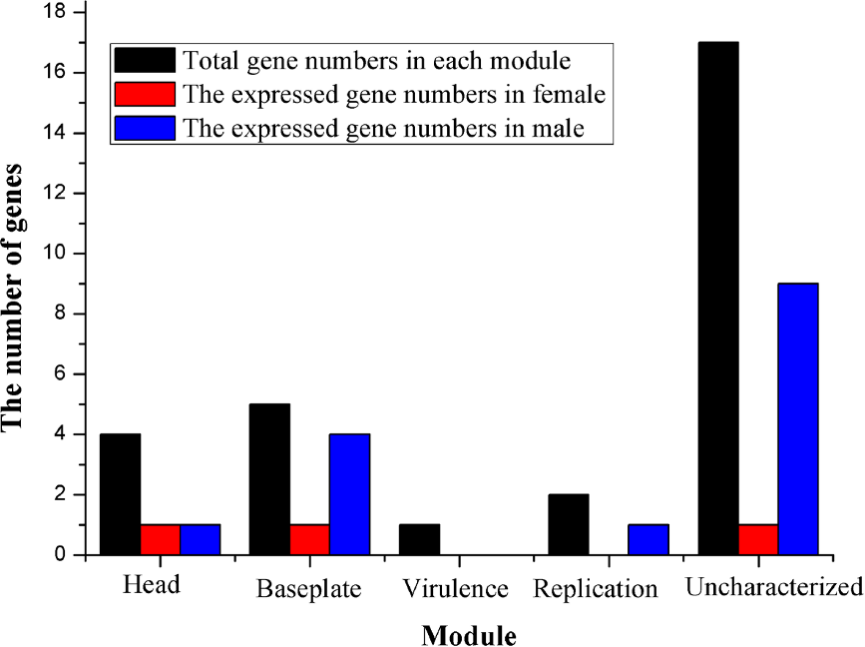
**Distributions of sex-specifically expressed ORFs in each module of the cryptic prophage WOSol.** In females, So0006 in the baseplate module, So0015 in the head module, and So0029 in the uncharacterized module are actively expressed; in males, So0005, So0007, So0008, and So0010 in the baseplate module, So0014 in the head module, So0024 in the replication module, and So0002, So0003, So0004, So0017, So0023, So0021, So0025, So0027, and So0029 in the uncharacterized module are actively expressed.

We then summarized the transcribed genes in the different modules of the WOSol genome. Of the three genes transcribed in females, So0006 and So0015 were from the baseplate and head module, respectively, but So0029 was uncharacterized. However, in males, all the modules, except the virulence module, included actively transcribed ORFs (Figure 2).

**Table 1:** **Sex-specific RNA expression of the cryptic prophage WOSol**

ORF ID	Product	♀	♂
So0001	Site-specific recombinase	n^-^	n^-^
So0002	Putative phage related protein	n^-^	+
So0003	Ankyrin repeat-containing prophage LambdaW1	n^-^	n^+^
So0004	Ankyrin repeat-containing prophage LambdaW1, authentic point mutation; This gene contains a premature stop which is not the result of sequencing error, pseudo	n^-^	n^+^
So0005	Tail I	n^-^	+
So0006	Prophage LambdaW1, baseplate assembly protein J	n^+^	n^-^
So0007	Prophage LambdaW1, baseplate assembly protein W	n^-^	+
So0008	Baseplate assembly protein GpV	n^-^	+
So0009	Conserved hypothetical protein	n^-^	n^-^
So0010	Prophage LambdaW5, minor tail protein Z, authentic frameshift; This gene contains a frame shift which is not the result of sequencing error, pseudo	n^-^	+
So0011	Conserved hypothetical protein	n^-^	n^-^
So0012	Major capsid protein, putative	n^-^	n^-^
So0013	Conserved hypothetical protein	n^-^	n^-^
So0014	Minor capsid protein C, putative	n^-^	+
So0015	Phage portal protein	+	n^-^
So0016	Lyzozyme M1	n^-^	n^-^
So0017	Conserved hypothetical protein	n^-^	n^+^
So0018	Phage terminase large subunit GpA, authentic frameshift; This gene contains a frame shift which is not the result of sequencing error, pseudo	n^-^	n^-^
So0019	Conserved hypothetical protein	n^-^	n^-^
So0020	Prophage LambdaW1, DNA methylase,authentic frameshift; This gene contains a frame shift which is not the result of sequencing error, pseudo	n^-^	n^-^
So0021	Putative Holliday junction resolvasome, endonuclease subunit	n^-^	+
So0022	RepA,fragement; This gene is a fragement which is not the result of sequencing error. Identified by similarity to NZ_CAGB01000010.1:4487..6541, pseudo	n^-^	n^-^
So0023	Putative rhoptry protein	n^-^	n^+^
So0024	Regulatory protein RepA, authentic frameshift; This gene contains a frame shift which is not the result of sequencing error, pseudo	n^-^	n^+^
So0025	Helicase, SNF2 family, authentic frameshift; This gene contains a frame shift which is not the result of sequencing error, pseudo	n^-^	n^+^
So0026	Patatin family protein, fragement; This gene is a fragement which is not the result of sequencing error. Identified by similarity to NC_002978.6:549882..550790, pseudo	n^-^	n^-^
So0027	Hypothetical protein	n^-^	n^+^
So0028	Ankyrin repeat protein	n^-^	n^-^
So0029	Ankyrin repeat protein	n^+^	+

**Notes:** +, positive using conventional RT-PCR; n^+^, positive using nested RT-PCR; n^-^, negative using both conventional and nested RT-PCR. **♂**, male adult of *C.solmsi*; ♀, female adult of *C.solmsi*.

The prophage genes showed variable expression levels and sex-specific differences in the fig wasp (Table 1). Of the expressed genes, some were highly expressed and could be detected with conventional RT–PCR, whereas some were expressed at low levels and could only be detected with an additional round of nested PCR. All of the actively transcribed genes were expressed in either the females or males, but not both, except an *ank* gene (So0029), which was actively transcribed in both females and males.

### Real-time qPCR assay of So0029 gene expression in female and male *C. solmsi*

**Figure 3:**
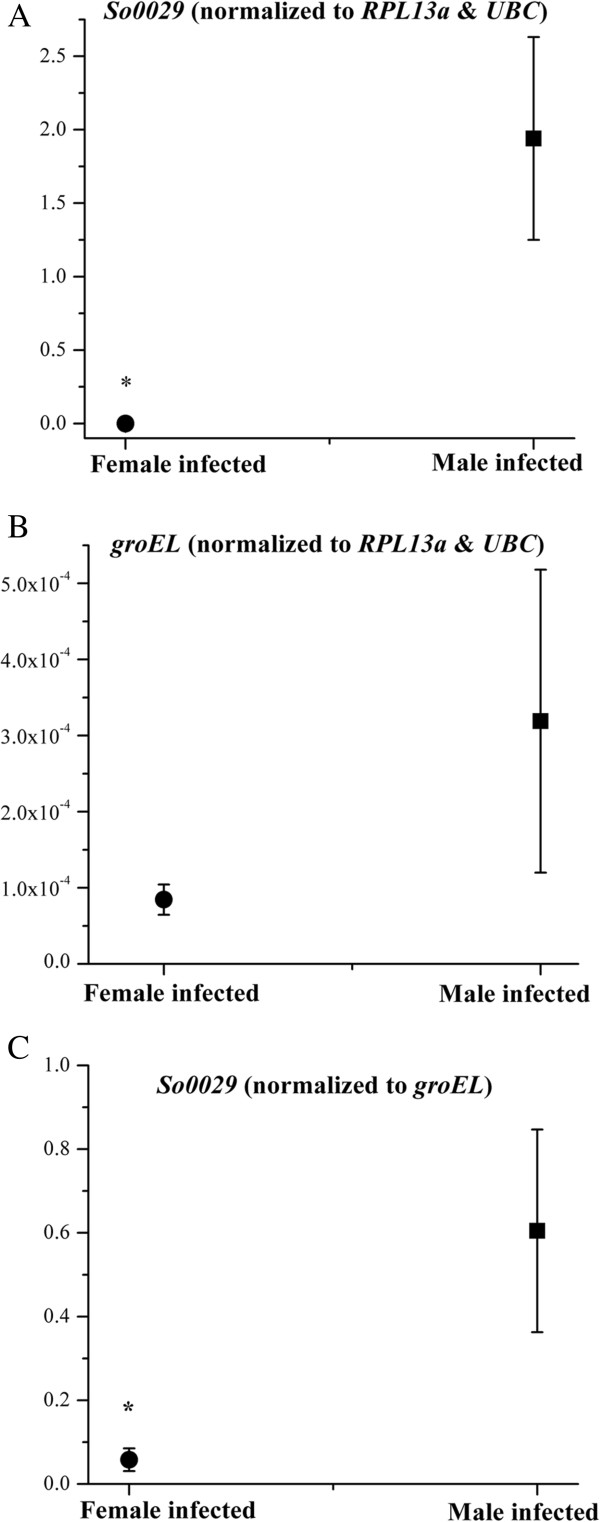
**Expressions of the** ***ank*** **gene in female and male individuals of** ***C. solmsi*** **.** Expression of So0029 was provided after normalized with fig wasp’s nuclear gene (*RPL13a & UBC*), and with *Wolbachia groEL* gene expression (**A** and **C**, respectively). Expression of *groEL* was provided relative to *RPL13a & UBC* expression **(B)**. Each value represents the average ± SE of six biological replicates for females and males. The asterisk indicates a significant difference between females and males (P <0.05).

The results described above showed that an *ank* gene (So0029), the only actively transcribed gene expressed in both females and males, differed in its expression in the two sexes: low in females and high in males (Table 1). However, these results were based on qualitative RT–PCR, and differences in primer sensitivity and primary template concentrations could affect the levels of amplified product. Therefore, we used real-time qPCR to quantitatively examine the expression of this gene (the primers are listed in Additional file 1). The So0029 gene was expressed with sex-dependent variations after normalization with the expression of the fig wasp’s nuclear genes of *RPL13a & UBC*, and *groEL* gene from *Wolbachia* (Figure 3A and C). However, the *Wolbachia* gene *groEL* showed sex-independent expression after normalization to the *RPL13a* and *UBC* genes, suggesting that the level of *Wolbachia* infection was not a major part of the observed variations of the phage gene So0029 (Figure 3B). Altogether, our data showed that the only one actively transcribed gene in both sexes, So0029, was sex-dependently expressed.

## Discussion

In this study, we confirmed the presence of a single cryptic prophage WOSol in the only *w*Sol genome in the fig wasp species *C. solmsi*, and demonstrated that a large proportion of the genes of the cryptic prophage were actively transcribed. Cryptic prophages are genetically defective because of the deletion or disruption of genes essential for their lytic growth and the production of infectious particles. Therefore, they have been regarded as simple genetic remnants, and researchers have tended to ignore their possible functions [29]. Recently, investigators noticed that cryptic prophages can confer multiple benefits on their hosts [27, 28]. However, how the cryptic prophage WO affects its host, *Wolbachia*, is poorly understood. To our knowledge, WORiA and WORiB are the only two known cryptic WO prophages confirmed by real-time qPCR to have no lytic processes, but have become trapped in the chromosome of *w*Ri [23]. Only three ORFs within the WORiB genome have been shown with RT–PCR to be actively transcribed and may therefore have roles in *Wolbachia* biology [20, 30]. These actively transcribed ORFs may function in *Wolbachia w*Ri during its infection of *D. simulans*, through prophage–prophage interactions, because *w*Ri harbors four prophage genomes [30]. Unexpectedly, in this study, we detected that of the 29 ORFs of the only cryptic prophage WOSol genome in *C. solmsi*, 17 are actively transcribed, which suggests that they may also play essential roles in the biology of *Wolbachia w*Sol. Moreover, some “cryptic” prophage haplotypes, although they have not been finally confirmed, have been reported to transcribe phage-related genes, with potential to affect the host biology. For example, the *met2* gene of “cryptic” prophages WOMelA and WOMelB within *w*Mel are actively transcribed in both sexes of *D. melanogaster* [20]. In the “cryptic” prophages within *w*Pip, the sex-specific expression of *ank* in *Culex quinquefasciatus* [33] and the stage-specific expression of *orf7* in *C. pipiens* [34] have also been detected. All these results suggest that in the cryptic prophage WO, there are many genes that play active roles to *Wolbachia* biology.

The phenomenon of the reproductive manipulation by *Wolbachia* of its host is compelling, but the molecular basis remains unknown [7, 35, 36]. One potential mechanism is the variable expression and activity of *Wolbachia* genes in the female and male insect hosts or their infections with different *Wolbachia* strains [37]. However, variable gene expression in *Wolbachia* is suggested to occur at a low rate, considering that only a small number of regulatory genes have been identified in the sequenced *Wolbachia* genomes [37, 38]. Interestingly, in bacteriophage WO, some genes are sex- [33, 34, 37], stage- [34], and strain-specifically expressed in the host [34, 39], which suggests that WO contributes to the manipulation by *Wolbachia* of its host. In the cryptic prophage WOSol, the expressions of ORFs are highly sex specific (three in female fig wasps, 15 in males, with only one ORF actively transcribed in both females and males), which leads us to make an assumption that these genes may have the possibility to be involved (directly or indirectly) in CI in *C. solmsi* by *w*Sol, based on the high prevalence of *Wolbachia* (more than 80% infection, as previously reported; 83.3% (364/437) in the present study) [31, 40] and the highly female-biased sex ratio of this species [40]. However, we need to experimentally confirm the present of CI phenotype in the species first. Furthermore, it is especially interesting that far more genes (15 of the 17 actively transcribed genes) are expressed in males, which further hints that the WO genes function as effectors, causing *Wolbachia* to exert different effects on the two sexes of the host. Future studies should examine the stage- and tissue-specific transcription of these phage ORFs. Interesting questions to be addressed are whether this sex-specific transcription reflects differences in the expression of the gene products in the ovaries and/or oocytes (in the female) and the testes and/or spermatocytes (in the male), and whether these genes may be involved in reproductive manipulation.

ANK mediates interactions between proteins, and thus acts as a transcription factor to regulate the expression of proteins involved in diverse aspects of cell biology [41, 42]. ANK is commonly found in eukaryote and viral proteins, whereas it is relatively rare in bacteria [41, 43]. There are often only 1–3 *ank* genes in the α-Proteobacteria, including *Rickettsia*, *Anaplasma*, and *Ehrlichia* [44, 45]. However, notably, some CI- inducing bacteria strains encode the largest number of ANK proteins. For example, there are 60 *ank* genes in *Wolbachia w*Pip from *C. pipiens* [46], 35 in *Wolbachia* strain *w*Ri infecting *D. simulans* [30], 23 in *Wolbachia* strain *w*Mel in *D. melanogaster* [47], and 19 in *Cardinium hertigii*, *c*Eper1 [48]. However, in mutualist *Wolbachia* strains, the *ank* genes are very reduced; for example, only five in *w*Bm [49] and six in *w*Oo [50]. The overrepresentation of ANK proteins in CI-inducing but distantly related *Cardinium* and *Wolbachia* strains thus suggests that ANK plays important roles in the process of CI [48]. The sequence variability of *ank* genes in CI-inducing strain *w*Mel and non-CI-inducing *w*Au [51], and sex-specific expression patterns of some *ank* genes in *w*Ri and *w*Pip also suggest that they function directly in the reproductive manipulation by the bacteria of their hosts [30, 33, 37]. We detect an *ank* gene that is actively transcribed in both the females and males of *C. solmsi*, and its level of expression is higher in males. Further investigation of all the *ank* genes in *Wolbachia w*Sol may help to determine whether ANK proteins are responsible for the reproductive manipulation of this fig wasp species by *Wolbachia*.

Surprisingly, we note that some structural prophage ORFs are actively transcribed in *C. solmsi*. For example, the ORF So0015 (in the head module) and So0006 (in the baseplate module) are actively transcribed in females, whereas ORF So0014 (in the head module) and ORFs So0005, So0007, and So0008 (all in the baseplate module) are actively transcribed in males. Structural ORFs are often expected to be expressed during the viral replication process and their transcription levels are considered to be evidence of whether bacteriophage WO is a lytic virion or an inactive prophage [34]. However, bacteriophage WOSol is a cryptic prophage and there is no viral replication. Therefore, rather than being actual structural/lytic genes responding to a density signal, these actively transcribed structural ORFs may have evolved some new functions in *C. solmsi*, distinct from their roles in viral structure formation.

## Conclusions

We comprehensively examine the transcription of a cryptic WO prophage in a *Wolbachia* strain and find that large proportion of the genes are actively expressed, which confirms that cryptic prophages are not nonfunctional fragments. The highly sex-specifically transcribed cryptic WO prophage genes may indicate their important roles in *Wolbachia* biology and its master manipulation of insect host, which need further study.

## Methods

### Sample collection

*Ceratosolen solmsi*, the pollinator species of *Ficus hispida* (Moraceae), was collected from Danzhou (N19°30′29″, E109°29′6″), Hainan Province, China, in June 2013. All fig fruits were collected at the same developmental stage, several days before becoming ripe. The female and male pollinators removed from the inside of the syconia were adults because the fig wasps are in the adult stage after they emerge from the galls into the fig syconia. They were identified and confirmed according to their morphological traits, under a Nikon SMZ80 microscope. Some specimens were immersed in Sample Protector (TaKaRa, Beijing, China) for RNA extraction and the others were immersed in 95% ethanol for DNA extraction.

In total, eight RNA sample groups (four female and four male samples; because the fig pollinators are very small, we used 10 whole-body individuals for each RNA sample) were collected to qualitatively determine the transcription of the prophage genes by RT–PCR. An additional 12 RNA sample groups (six female and six male samples; each sample contained 10 individual wasps) were collected to quantitatively determine the transcription of the So0029 and *groEL* genes with real-time qPCR.

DNA was extracted from 31 female and 35 male wasps to determine their infection with *Wolbachia*, and to compare the gene densities of *Wolbachia* (*w*Sol) and the WO prophage (WOSol), determined with real-time qPCR.

### RNA isolation and cDNA synthesis

Total RNA from each RNA sample was extracted with TRIzol Reagent (Invitrogen) and treated with RNase-free DNaseI (Invitrogen). A NanoDrop-2000 spectrophotometer (Thermo, Madison, WI, USA) was used to measure the RNA purity (A_260_/A_280_) and concentration. The key issue related to this method is the “false positives” generated by genomic DNA contamination, so before reverse transcription, all RNA samples were confirmed to contain no genomic DNA contamination by PCR with the universal *Wolbachia wsp* 81 F/691R primers [52] using TransTaq polymerase High Fidelity (TransGen Biotech, Beijing, China), [20, 34] (Additional file 5). First-stranded cDNA was then synthesized from 1 μg of total RNA with random primers [53] in a 20 μl reaction volume using TransScript II First-Strand cDNA Synthesis SuperMix (TransGen Biotech, Beijing, China). *wsp* expression was characterized as the positive control to demonstrate the quality of all the cDNA samples (Additional file 5).

### DNA extraction

Total genomic DNA was extracted from each wasp using the EasyPure Genomic DNA Extraction Kit (TransGen Biotech, Beijing, China), following the manufacturer’s recommendations, and suspended in 20 μl of double-distilled sterile water. DNA purity and concentration was determined with a NanoDrop-2000 Spectrophotometer (Thermo, Madison, WI, USA). The *Wolbachia* infection status of these wasps was confirmed by PCR with the *wsp* 81F/691R primers [52].

### RT–PCR and real-time qPCR expression analysis

Only expressed prophage genes can confer benefit on its bacterial host [54]. Therefore we tested the candidate functional genes for transcription. RT–PCR and sometimes nested RT–PCR with inner primer pairs were used to qualitatively determine the expression of all 29 ORFs of the prophage WOSol. Nested RT–PCR was only used when conventional RT–PCR did not detect the targeted fragment. Two samples were tested to represent each gene and sex; the positive results for the expressed genes that are presented in Table 1 show that all the genes were expressed in both samples.The resulting amplicons were run on a 1% TBE agarose gel and photographed under UV illumination. The PCR products were purified with the EasyPure PCR Purification Kit (TransGen Biotech, Beijing, China) and directly sequenced with an ABI3730 capillary autosequencer (Biosune, Beijing, China).

Real-time qPCR was performed with a Stratagene Mx3000p qPCR system (Stratagene, La Jolla, CA) (the primers are listed in Additional file 1). Reaction volumes of 20 μl containing 1 μl of template, 10 μl of TransStart Green qPCR SuperMix UDG (TransGen Biotech, Beijing, China), 0.4 μl of Passive Reference Dye II (50×) (TransGen Biotech, Beijing, China), 0.8 μl of primer mix (0.2 mM), and 7.8 μl of sterile water were prepared. A no-template control was included in each run to check for reagent contamination. A melting curve analysis was performed for each run to confirm the amplification specificity. The same thermal conditions were used for all real-time qPCR reactions: 40 cycles of 95°C for 10 s, 57°C for 15 s, and 72°C for 10 s. Two technical replicate experiments were performed for each real-time qPCR assay.

To quantify the densities of a minor capsid protein gene (*orf7*) from WOSol and a heat-shock protein 60 gene (*groEL*) [5] from *w*Sol in the DNA templates, we prepared standard solutions for the real-time qPCR. The PCR amplicons for *orf7* or *groEL* were resolved electrophoretically on TBE 1.0% agarose gel, and then cloned with the pEasy-T5 Zero Cloning Kit (TransGen Biotech, Beijing, China). The plasmids were then prepared with the EasyPure Plasmid MiniPrep Kit (TransGen Biotech, Beijing, China), and quantified with a NanoDrop-2000 spectrophotometer (Thermo, Madison, WI, USA). Standard 10-fold dilution series from 10^7^ to 10^3^ copies were prepared and used to calculate the copy numbers of the genes.

We also calculated the expression of So0003, So0007, So0014, So0015, and So0025 relative to that of reference genes. Several studies have demonstrated that the mean of individual PCR efficiencies (*E*_*m*_) gives a more reliable result than efficiencies derived with a standard curve because interwell differences would lead to the erroneous determination of gene expression, and an assumption of identical efficiency for each well confound the data analysis [55–57]. Therefore, we obtained all the *E*_*m*_ values by determining the baseline from the raw real-time qPCR data using LinRegPCR [58, 59]. The quantification cycle (*C*_*q*_) and *E*_*m*_ values obtained from LinRegPCR were then used to calculate the relative expression of the selected genes with respect to the reference genes *RPL13a* and *UBC*, with the following equation [60]:


*R*_*i*_ is the expression of each selected gene; *R*_*ref*_ is the expression of the reference genes; *R*_*i*_/*R*_*ref*_ is the expression of each selected gene normalized to that of the reference genes; Cq^*-i*^ and Em^*-i*^ are the quantification cycle value and the mean individual PCR efficiency for each selected gene, respectively; *Cq*^*_a*^ and *Cq*^*_b*^ are the quantification cycle values for each reference gene; *Em*^*_a*^ and *Em*^*_b*^ are the mean individual PCR efficiencies for each reference gene. Six biological replicates for females and males were performed in our experiments. Two technical replicate experiments were performed for each real-time qPCR assay.

### Statistical analysis

The average copy number of the integrated phage was compared with the expected number and the difference was analyzed statistically with a two-tailed *t* test (SAS Institute, Cary, NC, USA). In the real-time qPCR experiments, small plate effects (the apparent trends towards slightly elevated or reduced threshold cycle (C_t_) values for the same template DNA used for the standard curve between different plates) were common. We normalized the plate effects using a common threshold with a multiple experiment analysis (MxPro QPCR Software, Stratagene, La Jolla, CA). Correlation coefficients were calculated using nonparametric Spearman’s rho (JMP v.5.0, SAS Institute, Cary, NC, USA). One-way ANOVA (SAS Institute, Cary, NC, USA) was used to test for variations in the levels of *ank* mRNA between female and male *C. solmsi*. The significance level for all analyses was set at P <0.05.

### MIQE guidelines

For the real-time qPCR, we followed the Minimum Information for Publication of Quantitative Real-Time PCR Experiments (MIQE) guidelines [61] to increase the reliability and integrity of the results, and to promote experimental consistency and transparency between research laboratories. The MIQE checklist is provided in Additional file 6.

## Availability of supporting data

The data sets supporting the results of this article are available in the LabArchives repository, DOI:10.6070/H4S46PXM and https://mynotebook.labarchives.com/doi/NzY1NzUuMnw1ODkwNC81ODkwNC9Ob3RlYm9vay8yNzczMDg2MjMxfDE5NDM4My4y/10.6070/H4S46PXM.

## Electronic supplementary material

Additional file 1: **The primer pairs used for real-time qPCR analysis. Notes: Es(%),PCR reaction efficiency; R** ^**2**^ **, Pearson correlation coefficient.** (DOCX 22 KB)

Additional file 2: **Summary statistics for the Quantitative PCR.** (PDF 87 KB)

Additional file 3: **All the primers used for RT-PCR and nested RT-PCR.** (DOCX 19 KB)

Additional file 4: **The electrophoresis pictures of the RT-PCR and nested RT-PCR for all the studied genes. So0001 ~ So0029, the ORF IDs of prophage WOSol.** For the image of each gene: M, 100 bp DNA ladder; For each ORF, the first round of RT-PCR (first lane, for female sample; second lane, for male sample; third lane, positive control with gonomic DNA as template; fourth lane, negative control with distilled water as template); If the first and/or the second lane did not detect the targeted fragment, then the fifth and/or the sixth lane is nested RT-PCR with diluted products of the first and/or the second RT-PCR as template; The following two lanes are nested RT-PCR with diluted products of the third and fourth lane PCR products as template. (PDF 460 KB)

Additional file 5: **The electrophoresis pictures of PCR products of** ***wsp*** **gene with** ***wsp*** **81 F/691R primers.** PCR based on template of total RNA with DNaseI treatment but no reverse transcription (A) and first-stranded cDNA samples which were synthesized from 1 μg of total RNA with random primers in a 20 μl reaction volume using TransScript II First-Strand cDNA Synthesis SuperMix (TransGen Biotech, Beijing, China) (B). The comparison between (A) and (B) indicates that the RNA samples are not contaminated by genomic DNA. Lane 1 ~ 19: the results of 19 samples. PC: positive controls with genomic DNA as template. NC: negative controls with distilled water as template. M: 100 bp DNA ladder. (PDF 114 KB)

Additional file 6: **Minimum Information for Publication of Quantitative Real-Time PCR Experiments.** (DOCX 20 KB)

## Abbreviations

ORFs: Open reading frames
*ank*: ankyrin-domain-containing
CI: Cytoplasmic incompatibility
*met2*: DNA adenine methyltransferase gene
real-time qPCR: real-time quantitative PCR
RT–PCR: Reverse transcription PCR
*Em*: Individual PCR efficiencies
*Cq*: Quantification cycle
Ct: Threshold cycle
MIQE: Minimum information for publication of quantitative real-time PCR experiments.
