# Supplementary material for: Large proportion of genes in one cryptic WO prophage genome are actively and sex-specifically transcribed in a fig wasp species
Source: BMC Genomics. 2014 Oct 13;15(1):893. doi: 10.1186/1471-2164-15-893 (PMC4201733; doi:10.1186/1471-2164-15-893)
Supplement: Supplementary file 1 — Additional file 1: The primer pairs used for real-time qPCR analysis. Notes: Es(%),PCR reaction efficiency; R 2 , Pearson correlation coefficient. (DOCX 22 KB) [file 12864_2014_6559_MOESM1_ESM.docx]

**The primer pairs used for real-time qPCR analysis.**

| Gene | Forward primer (5’-3’) | | Reverse primer (5’-3’) | Amplicon length (bp) | Es(%) | R^2^ | Reference |
| --- | --- | --- | --- | --- | --- | --- | --- |
| *RPL13a* | | CTGCTCGTGGTCCTTTCCATTTTC | GCATCCTTGCCTCTTTGTGTCTTG | 97 | 104.4 | 0.997 | [[1](#_ENREF_1)] |
| *UBC* | GAAGCGGATCAACAAGGAACT | | GGACTGTCAGGTGGACCCATAAT | 123 | 96.6 | 0.992 | [[1](#_ENREF_1)] |
| *ank* | AAAGCAAATGTTCATCTGA | | AAGGTTATTCCGTAAAGC | 122 | 103.6 | 0.989 | This study |
| *groEL* | CAACCTTTACTTCCTATTCTTG | | CTAAAGTGCTTAATGCTTCACCTTC | 97 | 99.0 | 0.990 | [[2](#_ENREF_2)] |
| *orf7* | CAAATAATAAAGGACCGTTGTAGC | | CAGGAGAAAGCGTATGGAGAAAT | 150 | 101.1 | 0.996 | This study |

**Notes:** Es(%)，PCR reaction efficiency; R^2^, Pearson correlation coefficient.

**References**

1. Wang B, Xiao JH, Bian SN, Niu LM, Murphy RW, Huang DW: **Evolution and expression plasticity of opsin genes in a fig pollinator, *Ceratosolen solmsi***. *PLoS ONE* 2013, **8**(1):e53907.

2. Bordenstein SR, Marshall ML, Fry AJ, Kim U, Wernegreen JJ: **The tripartite associations between bacteriophage, *Wolbachia*, and arthropods**. *PLoS Pathog* 2006, **2**(5):e43.
