# Supplementary material for: Large proportion of genes in one cryptic WO prophage genome are actively and sex-specifically transcribed in a fig wasp species
Source: BMC Genomics. 2014 Oct 13;15(1):893. doi: 10.1186/1471-2164-15-893 (PMC4201733; doi:10.1186/1471-2164-15-893)
Supplement: Supplementary file 2 — Additional file 2: Summary statistics for the Quantitative PCR. (PDF 87 KB) [file 12864_2014_6559_MOESM2_ESM.pdf]

**Summary statistics for the Quantitative PCR.**

| Strain | Number | Mean Copy Number ( $\times 10^3$ ) ±SE | | Mean Density ± SE |
|---|---|---|---|---|
| | | *Wolbachia groEL* | Phage WOSol *orf7* | *orf7:groEL* |
| Female | 31 | 2.20 ±0.42 | 1.69 ±0.30 | 0.88 ±0.05 |
| Male | 35 | 6.30 ±1.09 | 6.43 ±1.03 | 1.15 ±0.06 |
