# Supplementary material for: Large proportion of genes in one cryptic WO prophage genome are actively and sex-specifically transcribed in a fig wasp species
Source: BMC Genomics. 2014 Oct 13;15(1):893. doi: 10.1186/1471-2164-15-893 (PMC4201733; doi:10.1186/1471-2164-15-893)
Supplement: Supplementary file 3 — Additional file 3: All the primers used for RT-PCR and nested RT-PCR. (DOCX 19 KB) [file 12864_2014_6559_MOESM3_ESM.docx]

**All the primers used for RT-PCR and nested RT-PCR.**

|  | RT-PCR primers | | Nested RT-PCR primers | |
| --- | --- | --- | --- | --- |
| ORF ID | Forward  (5’-3’) | Reverse (5’-3’) | Forward (5’-3’) | Reverse  (5’-3’) |
| So0001 | ACATCATAGCCGAGTGGTGGA | AAGCCGAGAAGGGTGGGT | TTCCTCCCATCCATAATCC | TTTGTAGCAGTGACGCAAAT |
| So0002 | CAAATAATAAAGGACCGTTGTAGC | CAGGAGAAAGCGTATGGAGAAAT | CGCTGATAATGACGTATAAAA | CAAGAAAAGGGGCAAAA |
| So0003 | CACCTGCTGTCGTACTTTCCG | GACGATATTACATTATGCAGTAGGGA | ATTTCTCGCATAATCCATTG | GGGAGTAGAGGTAAACGTAGTG |
| So0004 | CCACCATTTACCACCTGC | GCTTGGAATGCGTCTGG | CTCGCTTATCTATTCATCACTT | GAAGATGGAAATACGGCAC |
| So0005 | CCCTGAATAATCCGATAACA | TGCATTGGGTAACAGACAAA | AAAAGCCTCATTAGCCTCG | GCCACCCGGTAAACACT |
| So0006 | CGCATCCTTTACTCTTCTATCT | ATTGAGCCACTGAACTTTGA | CCTCCAGTGCTCCAGCCAACT | GAGAAAGAATAAACGAAGCGGTAAA |
| So0007 | ATACCACAATCCCACCA | ACACCGATAGGATCACG | ATACCACAATCCCACCAA | GAAATCTATGCAGCAGTAGC |
| So0008 | CTTGATTCCCCAACTTTT | GGGTAACGGCAAGAGC | AACTTTGTCCCATCTTGAA | GAGTTGTCATTAGGGGTAGT |
| So0009 | CCCATTCAACCATCCACA | GAAATACCAGCGATACAGACAT | TCTCACAACTACCGAGGAATC | ATAAGAAGGGAAATAGTAGCACC |
| So0010 | GCTTCTGGCTCCAATGCTAA | TTTGTCGGGGAGATGGTTT | GTTTCATCTTGCCAACTGC | CATAGAAATCACCGAAAGCA |
| So0011 | GCATCCTGACGCCTGAA | GCAGCAAGCGTTGTATGAA | TGAATTATCCCTAAGTGGTGG | CATACATGGTTCAGGTGCTG |
| So0012 | TATCCTCGGACGGTGGC | GTTAAGCGTAAGTGTCTGGAAGTA | TATCCTCGGACGGTGGC | ACCTTCATACCCAATCAAACC |
| So0013 | TGATGCCCCGTGCT | CGGTAAAACTAAAGGGAAAA | AATGATTACTGCTTTGGTGGTT | TGGTACGGTTGTTGGTCGT |
| So0014 | CTTTCCTGCAAATACTGTGG | CTGGCTCAATAAACCGATG | GCTTCGTAAATAAAGTCCG | AGAAATAGTGAAAGGGGAAT |
| So0015 | GTTCCAGGTTCTAAACCAGATAA | AGCAGACAGTAGCGGAGTAAGT | CCCCTTGCTCATTGCTTT | GAGGAGTACCGTGGCTTTCT |
| So0016 | CACATGAGCCTATTCCTTCTAC | CACCAGGGATGCTTGTAAAT | ACGTCCCGTTACTACATAAACTC | GCGGCAAAGACCAAGC |
| So0017 | CACCTGCGACTTTCAAT | ACTCAAGTAGAAGAAGCGATA | CTTCTCCATACCGAACTACA | AGAGCGGGGAAAGAGTA |
| So0018 | ATCTACCCAGGTTTCTCCGAGTG | GCAAGAGTTGTCCGTAATAATTTGCT | GTAGTGGGCTGTTGAGGGA | GAAATGGGCAAGCGTTG |
| So0019 | TGCTAGTAATGCTGAAACCCTA | CAAACAGAATGGGCAAGG | CCCCGCTCCATCTCGTT | TTGCCAAGCAATATGTCTGTTA |
| So0020 | TTCCAGTATCAGCGAGAATAGCAT | GTGACCCTCCGTATAACGTAGCA | CCCTGGTCCACTGCTG | GATTATCAAAGACAATACGAAAC |
| So0021 | AGCATCAACGCCAAAGC | CCTCGGCAAGCAAACC | TAGAAAGCCTCCGTAGCA | TGAAGCAGTGTATTTTGAAGAA |
| So0022 | ATAGCGTCCGTTCATCTTT | AGTTTACCCCTTTCTTTCAT | CATGGACAAGTGCCAGACT | GGATTTATTTGGTGGAGACC |
| So0023 | CCTCAAATAGGGCATAGC | TAATCCGCTTAATGGAGTAG | TTCTGGCTGGCGTATAA | AAAACAGTTGGATGAGTTAGAT |
| So0024 | CACCTGCTTCATCATTATCTGGC | AGACCCCAATCAAAACAGC | TAACATCTGCGTTTGCTCC | TGATGATAGTGGAAAGCGATA |
| So0025 | CAAGGCATAGCCCAATC | TTATGATAACCGTCTTCCAC | TTTGACGAATGGTCTGTTG | GTGGCAGAACTTGTGCTAATA |
| So0026 | CACAACTAAAAGTAGCATCAG | TTTGCTTGGCATTTCA | CACAACTAAAAGTAGCATCAG | TTGCATTTGCCTCCTG |
| So0027 | AAAGCAAAACCCGTAGA | TCTTTACTTACTGGTTTTATAGAC | AAACAAGATAATCGGGAGC | AGTTATGGCTAAGGCTGGA |
| So0028 | TCAAACATGGTGCTAACG | CTTCGCAATAAAAGCTCTAC | GTTGCTTCCATATTTGGCTTTA | GCGCCTCCCTTTATCAGTA |
| So0029 | AAAGCAAATGTTCATCTGA | GGTTATTCCGTAAAGCAG | TTATAACTCTAAAGAAGGTTACAC | CGTTTTCATCCGCTACA |
