# Supplementary figures and images for: Large proportion of genes in one cryptic WO prophage genome are actively and sex-specifically transcribed in a fig wasp species
Source: BMC Genomics. 2014 Oct 13;15(1):893. doi: 10.1186/1471-2164-15-893 (PMC4201733; doi:10.1186/1471-2164-15-893)

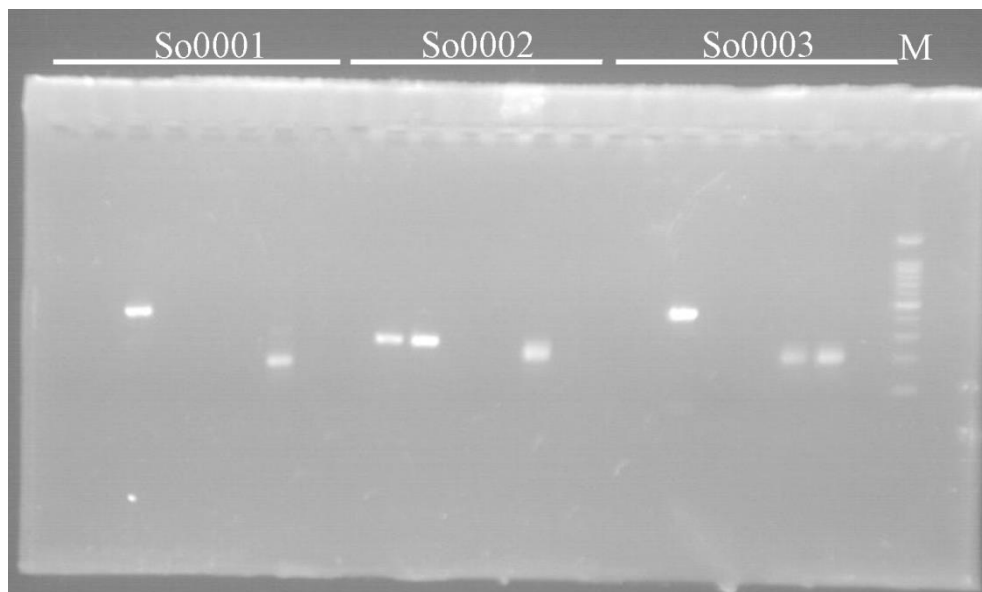

1

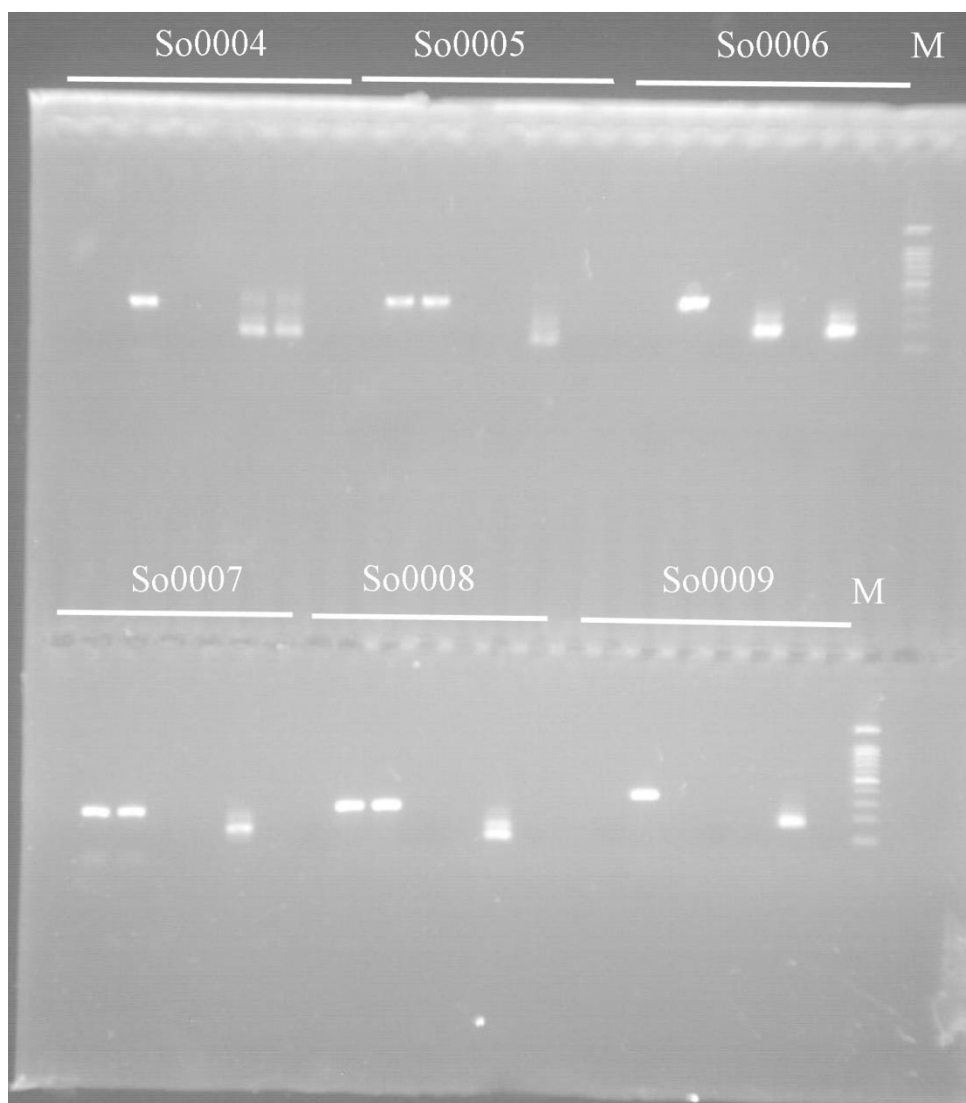

2

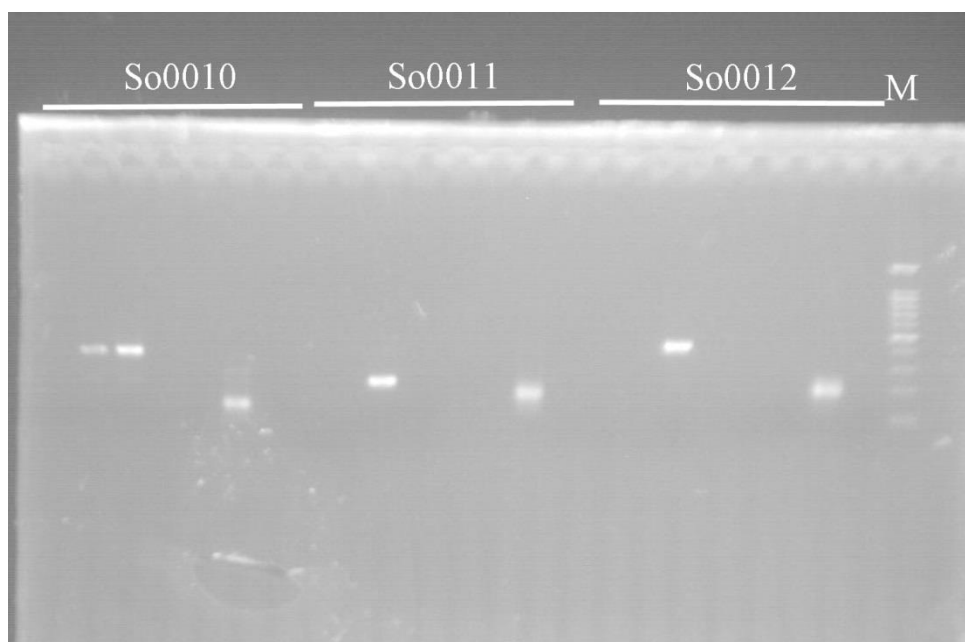

3

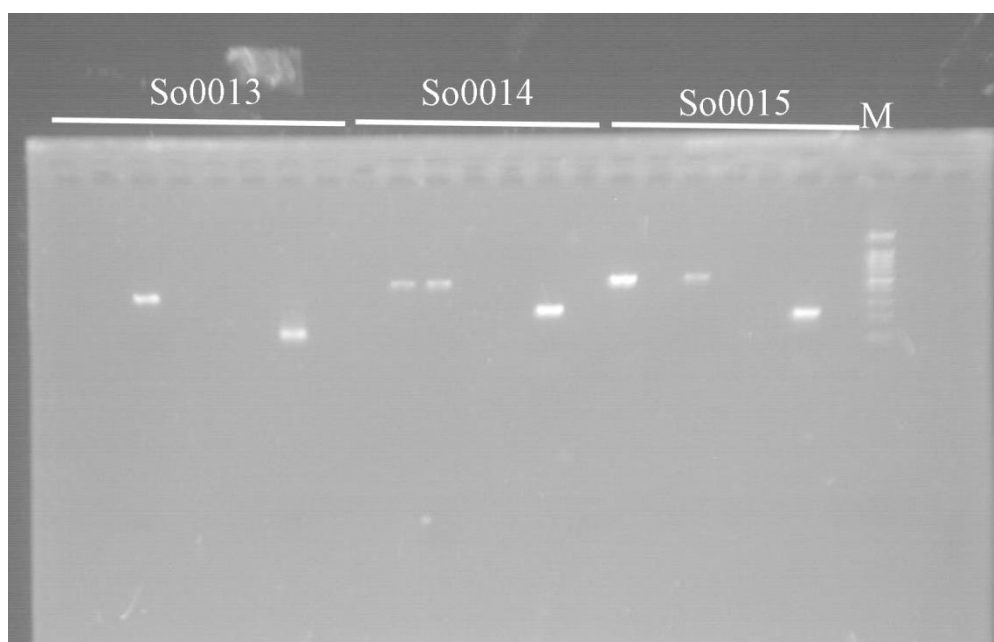

4

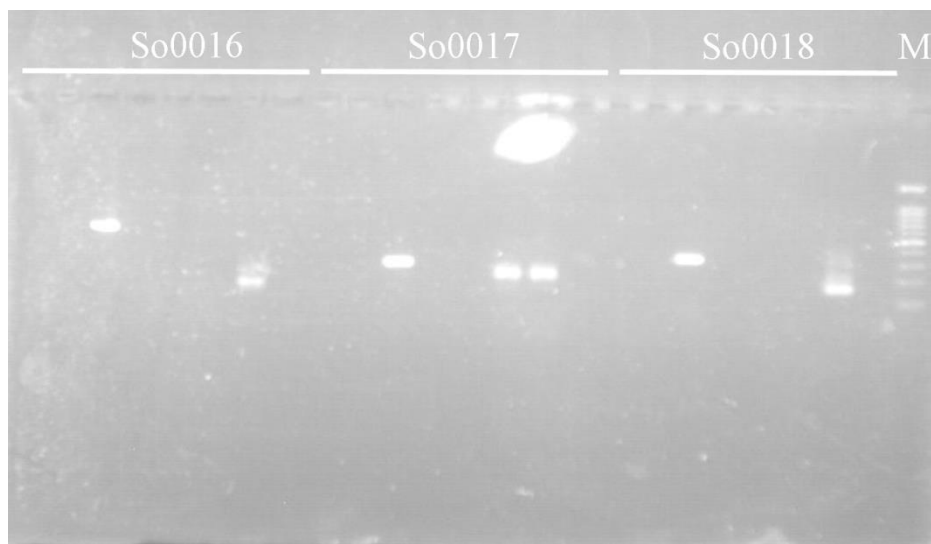

5

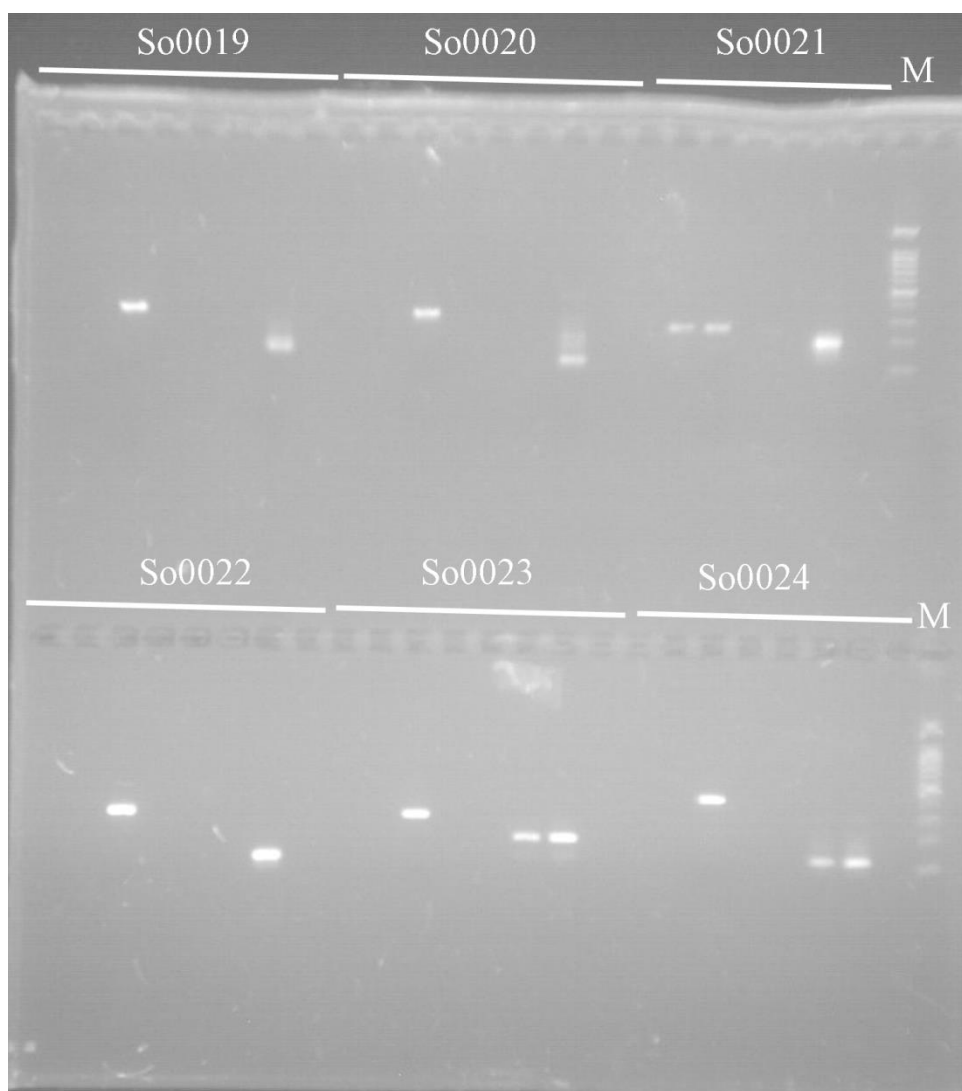

6

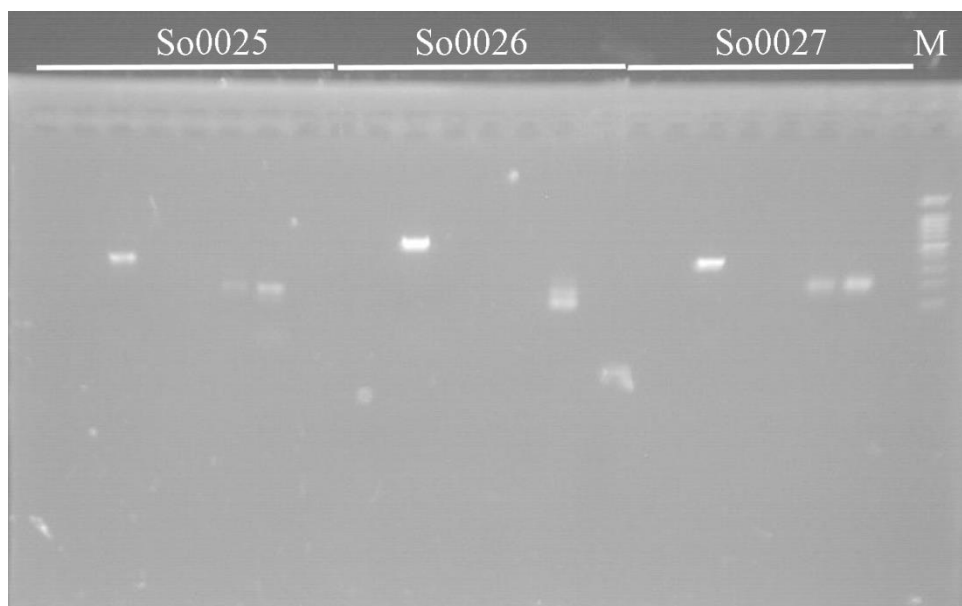

7

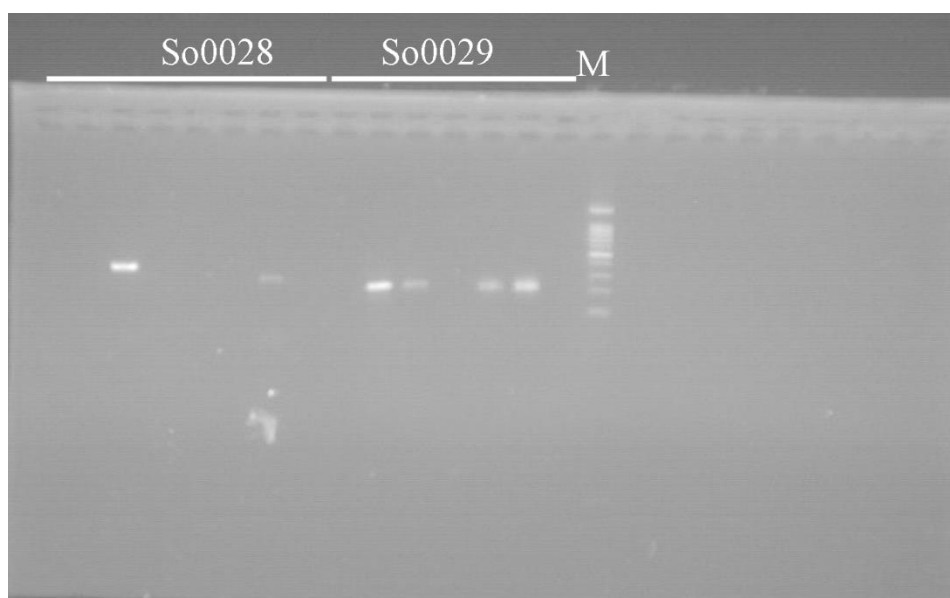

8

9

Supplement: Supplementary file 4 — Additional file 4: The electrophoresis pictures of the RT-PCR and nested RT-PCR for all the studied genes. So0001 ~ So0029, the ORF IDs of prophage WOSol. For the image of each gene: M, 100 bp DNA ladder; For each ORF, the first round of RT-PCR (first lane, for female sample; second lane, for male sample; third lane, positive control with gonomic DNA as template; fourth lane, negative control with distilled water as template); If the first and/or the second lane did not detect the targeted fragment, then the fifth and/or the sixth lane is nested RT-PCR with diluted products of the first and/or the second RT-PCR as template; The following two lanes are nested RT-PCR with diluted products of the third and fourth lane PCR products as template. (PDF 460 KB) [file 12864_2014_6559_MOESM4_ESM.pdf]

1

2 A

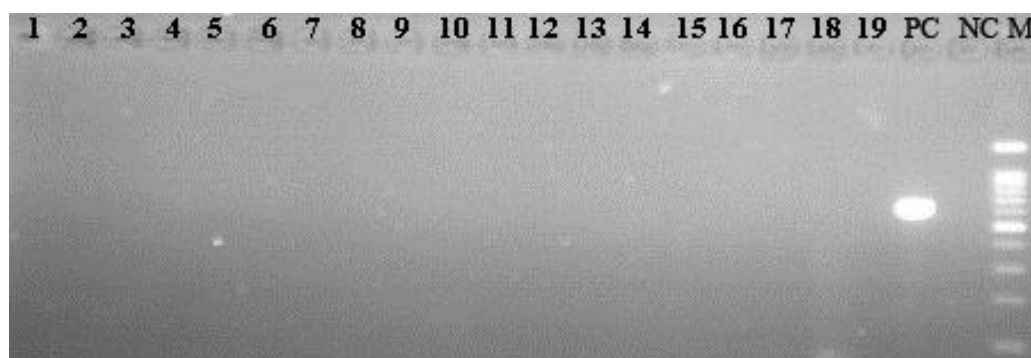

3

4

5 B

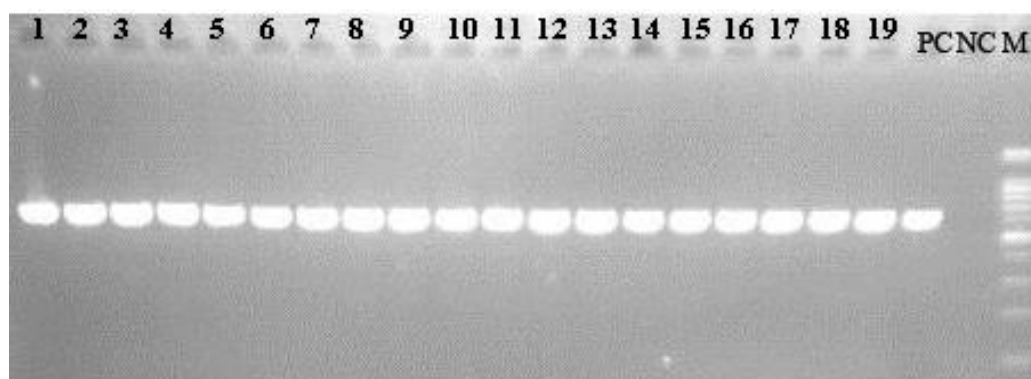

6

7

Supplement: Supplementary file 5 — Additional file 5: The electrophoresis pictures of PCR products of wsp gene with wsp 81 F/691R primers. PCR based on template of total RNA with DNaseI treatment but no reverse transcription (A) and first-stranded cDNA samples which were synthesized from 1 μg of total RNA with random primers in a 20 μl reaction volume using TransScript II First-Strand cDNA Synthesis SuperMix (TransGen Biotech, Beijing, China) (B). The comparison between (A) and (B) indicates that the RNA samples are not contaminated by genomic DNA. Lane 1 ~ 19: the results of 19 samples. PC: positive controls with genomic DNA as template. NC: negative controls with distilled water as template. M: 100 bp DNA ladder. (PDF 114 KB) [file 12864_2014_6559_MOESM5_ESM.pdf]
