# Supplementary material for: Large proportion of genes in one cryptic WO prophage genome are actively and sex-specifically transcribed in a fig wasp species
Source: BMC Genomics. 2014 Oct 13;15(1):893. doi: 10.1186/1471-2164-15-893 (PMC4201733; doi:10.1186/1471-2164-15-893)
Supplement: Supplementary file 6 — Additional file 6: Minimum Information for Publication of Quantitative Real-Time PCR Experiments. (DOCX 20 KB) [file 12864_2014_6559_MOESM6_ESM.docx]

**Minimum Information for Publication of Quantitative Real-Time PCR Experiments.**

| **Item to check** | **Importance** | **Details** |
| --- | --- | --- |
| **Sample Description** | Essential | Pollinators (*Ceratosolen solmsi*) of *Ficus hispida* |
| **If frozen, how and how quickly** | Essential | Insect samples were immediately frozen in liquid nitrogen after they were collected |
| **If fixed, with what and how quickly?** | Essential | Stored in sample Protector (TAKARA, China) immediately after frozen |
| **Sample storage conditions and duration** | Essential | Samples were held at -80 ^o^C for less than a week before RNA isolation |
| **Experimental design** |  |  |
| **Definition of experimental and control groups** | Essential | No relative quantification were involved in this work, thus no control groups were defined |
| **Number within each group** | Essential | *orf7, Grol*: (female 31, male 35); *ANK, UBC, RPL13a* (female 5, male 6) |
| **Nucleic acid extraction** |  |  |
| **Procedure and/or instrumentation** | Essential | For each RNA sample, total RNA of 8 individuals was extracted by using TRIzol (Invitrogen) |
| **Name of kit and details of any modifications** | Essential | EasyPureTM RNA kit (Transgen, China). We exactly followed the protocols of the kit |
| **Details of DNase or RNase treatment** | Essential | Genomic DNA was removed by treating with DNaseI (Invitrogen) according to the standard protocols |
| **Contamination assessment (DNA or RNA)** | Essential | No template control (NTC) was performed for each sample to assess contamination. |
| **Nucleic acid quantification** | Essential | RNA concentration was determined by measuring the abosorbance at 260nm UV light |
| **Instrument and method** | Essential | NanoDrop-2000 Spectrophotometer (Thermo, USA) |
| **RNA integrity: method/instrument** | Essential | RNA integrity was assessed by electrophoresis on 1.0% agarose gels stained with ethidium bromide |
| **RIN/RQI or C_q_ OF 3’ and 5’ transcripts** | Essential | N/A |
| **Inhibition testing (C_q_ dilutions, spike, or other)** | Essential | Standard curve analyses were sufficient to test inhibition |
| **Reverse transcription** |  |  |
| **Complete reaction conditions** | Essential | TransScript II First-Strand cDNA Synthesis SuperMix (Transgen, China) was used to generate single-stranded cDNA total RNA with random primers. |
| **Amount of RNA and reaction volume** | Essential | Amount of RNA: 1μg; Reaction volume: 20μl |
| **Priming oligonucleotide and concentration** | Essential | random primers: 2μM |
| **Temperature and time** | Essential | 25^o^C for 10 minutes, 42^o^C for 30 minutes, and85^o^C for 5minutes, |
| **qPCR protocol** |  |  |
| **Complete reaction conditions** | Essential | PCR reactions were performed in a Mx3000P Real Time Thermocycler (Stratagene). A 20 μl PCR mixture was prepared containing 1 μl of template, 10μl TransStart Green qPCR SuperMix UDG(2x) (Transgen, China), 0.4μl Passive Reference Dye II(50x) (Transgen, China), 0.8μl primer mix(0.2μM), and 7.8 μl sterile water. The following thermal conditions for qRT-qPCR were used: 50^o^C for 2 min, 95^o^C for 10 min, and then the follwing: 95^o^C for 10 s, 57^o^C for 15 s and 72^o^C for 10 s for 40 cycles |
| **Reaction volume and amount of cDNA/DNA** | Essential | Reaction volume: 20μl; amount of cDNA: 1μl per reaction volume |
| **Primer, (probe), Mg2, and dNTP concentrations** | Essential | 500nM primers; 3mM MgCl_2_ ; 0.2 mM dNTP |
| **Polymerase identity and concentration** | Essential | TransStart Green qPCR SuperMix UDG (2x) (Transgen, China) |
| **Buffer/kit identity and manufacturer** | Essential | TransStart Green qPCR SuperMix UDG (2x) (Transgen, China) |
| **Additives (SYBR Green I, DMSO, and so forth)** | Essential | Passive Reference Dye II(50x) (Transgen, China) |
| **Complete thermocycling parameters** | Essential | 50^o^C for 2 min, 95^o^C for 10 min, and then the follwing: 95^o^C for 10 s, 57^o^C for 15 s and 72^o^C for 10 s for 40 cycles |
| **Specificity (gel, sequence, melt, or digest)** | Essential | Melting curve analysis, gel electrophoresis and sequencing |
